# 1411. Noninvasive Assessment of Intralesional Antimicrobial Concentration-Time Profiles in Pulmonary and Central Nervous System Tuberculosis using Dynamic ^18^F-Pretomanid Positron Emission Tomography

**DOI:** 10.1093/ofid/ofab466.1603

**Published:** 2021-12-04

**Authors:** Filipa Mota, Camilo Ruiz-Bedoya, Elizabeth Tucker, Patricia De Jesus, Kelly Flavahan, Mitchell Turner, Clara Erice, Melissa Bahr, John Kim, Farina Mahmud, Charles A Peloquin, Charles A Peloquin, Alvaro A Ordonez, Sanjay K Jain, Sanjay K Jain

**Affiliations:** 1 Johns Hopkins, Baltimore, Maryland; 2 University of Florida, Gainesville, FL

## Abstract

**Background:**

Pretomanid is used in combination with bedaquiline and linezolid (BPaL regimen) in the treatment of multidrug-resistant tuberculosis (MDR-TB). However, the penetration of pretomanid in privileged sites remain unknown. Antimicrobial pharmacokinetic (PK) parameters are traditionally derived from clinical samples (blood and cerebrospinal fluid), which may not accurately represent the intralesional tissue PK, affected by drug properties, vascular supply, barrier permeability, and the microenvironment.

**Methods:**

We developed ^18^F-pretomanid (chemically identical to pretomanid) for *in vivo* multi-compartment PK by positron emission tomography (PET). Dynamic ^18^F-pretomanid PET was used to obtain cross species pretomanid concentration-time profiles in animal models of TB (mice and rabbits) to quantify penetration into pulmonary and brain lesions. A subset of animals underwent PET/CT imaging with ^18^F-py-albumin and ^18^F-FDG to assess vascular supply and inflammation. Postmortem ^18^F-pretomanid autoradiography (high-resolution) and mass spectrometry were performed in infected tissues. A mouse model of TB meningitis was used to evaluate the bactericidal activity of the BPaL regimen (Figure 1).

Figure 1. Experimental schematics.

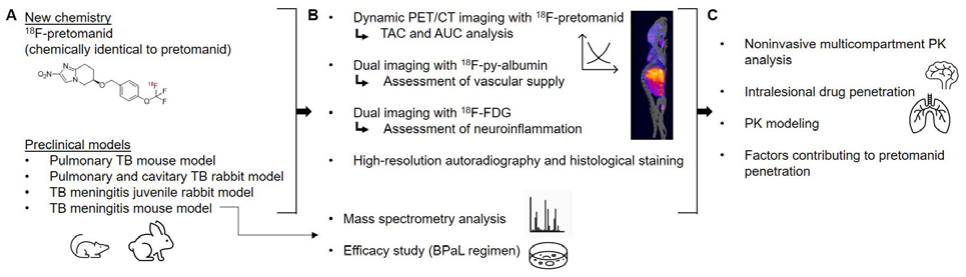

(A) A new synthetic approach was developed to obtain radiofluorinated pretomanid (18F-pretomanid), which is chemically identical to pretomanid and therefore undergoes identical PK and metabolism in vivo. Dynamic 18F-pretomanid PET/CT imaging was performed in validated preclinical models of tuberculosis following intravenous administration of 18F-pretomanid. (B) PET signal was quantified in multiple compartments to generate time activity curves (TACs) used to calculate area under the curve (AUC) over 0-60 minutes. A subset of animals also underwent PET/CT imaging of 18F-py-albumin to assess vascular supply to lung and brain lesions, and with 18F-FDG to confirm the presence of neuroinflammation in the mouse and rabbit models of TB meningitis. Tissue resection post-mortem was used to visualize the intralesional retention of 18F-pretomanid using high-resolution (10 µm) autoradiography. The efficacy of the BPaL regimen in TB meningitis was compared to that of standard treatment with rifampin, isoniazid, and pyrazinamide in the mouse model. Mass spectrometry was performed following oral administration of BPaL to determine brain drug levels. (C) These data provide multicompartment PK analysis, intralesional levels of pretomanid, and insights into the mechanism that govern pretomanid tissue distribution.

**Results:**

^18^F-Pretomanid PET provided detailed concentration-time profiles in infected tissues demonstrating excellent lung and brain tissue penetration (AUC ratio to plasma > 1) in both animal species, which was spatially compartmentalized, likely due to differential vascular supply (^18^F-py-albumin PET) (Figure 2). Brain lesions (identified by ^18^F-FDG PET) demonstrated localized leakiness on ^18^F-py-albumin PET. Autoradiography and mass spectrometry corroborated the imaging findings. The efficacy of the BPaL regimen in TB meningitis was substantially lower than standard TB treatment (Figure 3), likely due to restricted penetration of bedaquiline and linezolid into the brain parenchyma.

Figure 2. Spatial heterogeneity of 18F-Pretomanid penetration and vascular supply to pulmonary TB lesions.

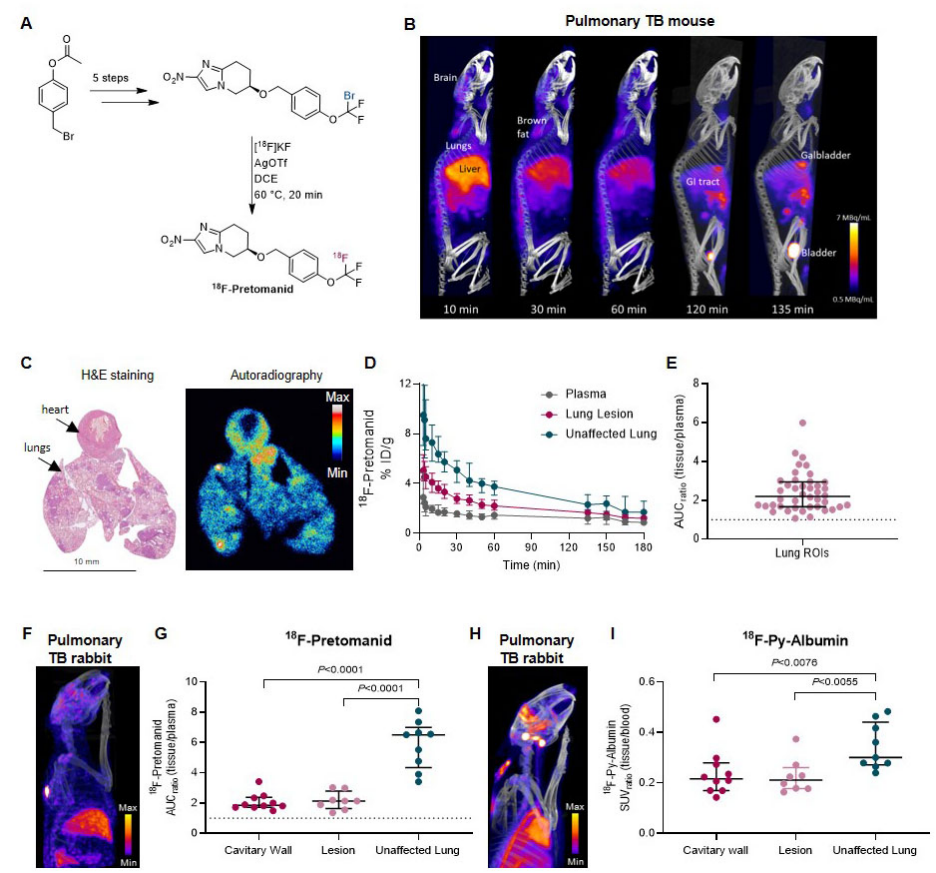

(A) A novel synthetic was devised to obtain 18F-pretomanid, which is chemically identical to pretomanid. (B) Maximum intensity projection (MIP) of 18F-Pretomanid PET/CT in M.tb.-infected mice over 3 hrs shows hepatobiliary and renal excretion, high uptake into brown fat, brain, and lungs. (C) Resection of infected lungs 30 minutes post intravenous administration of 18F-pretomanid shows heterogenous distribution of 18F-pretomanid into the lungs visible by high resolution autoradiography. Areas of pneumonia are identifiable by hematoxylin and eosin (H&E) staining of the same tissue section used for autoradiography. (D) Time-activity curves of 18F-Pretomanid in infected mouse lung (0-3 hours) and derived area under the curve (AUC) ratios to plasma (E) in infected mouse lung. Representative MIP of 18F-pretomanid (F) and 18F-py-albumin (H) PET/CT in a rabbit with cavitary TB and quantification of the AUC ratios to plasma show reduced penetration into lung lesions and cavitary wall compared to areas of unaffected lung (G and I). Data are represented as median ± interquartile range, n=3-4 group.

Figure 3. Exposure levels of 18/19F-pretomanid in models of TB meningitis.

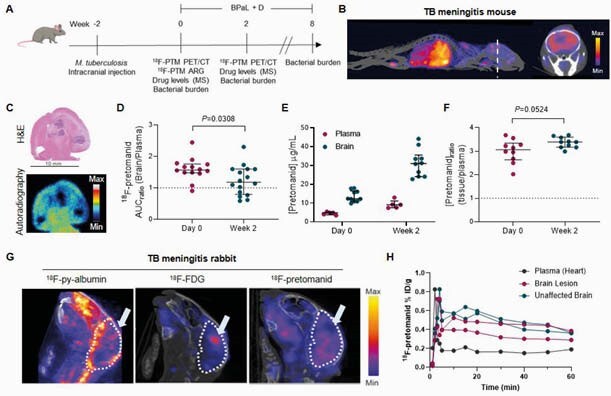

(A) Experimental timeline used to assess the penetration of pretomanid into infected mouse brain before and during treatment with antimicrobials bedaquiline (B), pretomanid (Pa), and linezolid (L), and corticosteroid dexamethasone (D). (B) Representative three-dimensional MIP of 18F-pretomanid PET/CT in the CNS-TB model, 10 min post-injection, and transverse section showing high and heterogeneous brain uptake. (C) High-resolution autoradiography was performed to confirm heterogeneous penetration of 18F-pretomanid into infected brain lesions in the mouse. (D). 8F-pretomanid AUC ratios of tissue to plasma in mouse brain before (day 0) and two weeks into treatment show a reduction in penetration at week 2. (E). Pretomanid concentrations (µg/mL) in mouse plasma and brain, at day 0 and two weeks into treatment, measured by mass spectrometry and derived concentration ratios of brain to plasma (F) suggest drug accumulation due to the long half-life. (G) While 18F-py-albumin and 18F-FDG PET/CT show vascular leakage and neuroinflammation in the rabbit model of TB meningitis, the penetration of 18F-pretomanid is heterogeneous and reduced at the lesion site (indicated by white arrow). (H) Quantification of the PET signal shows variability within the same animal. Data are represented as median ± interquartile range, n=3-5 group.

Figure 4. Evaluation of a pretomanid-containing regimen in TB meningitis.

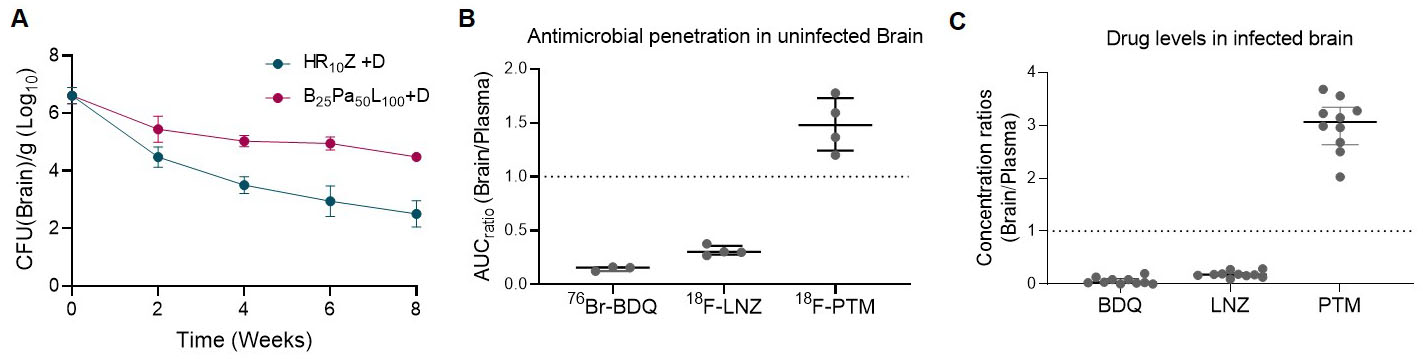

(A) Mice with experimentally induced TB meningitis were treated with Bedaquiline (25 mg/day), Pretomanid (100 mg/day), Linezolid (100 mg/day), and Dexamethasone (2 mg/day) or Rifampin (10 mg/day), Isoniazid (10 mg/day), Pyrazinamide (150 mg/day) and Dexamethasone (2mg/day) for 8 weeks. Treatment efficacy was determined based on the brain bacterial burden after 2, 4, 6, and 8 weeks of treatment. (B) The penetration of 76Br-bedaquiline, 18F-linezolid, and 18F-pretomanid into the brain parenchyma was measured non-invasively by PET and revealed low penetration of 76Br-bedaquiline (AUC radio to plasma 0.15) and 18F-linezolid (AUC radio to plasma 0.3). (C) Mass spectrometry analysis was performed to confirm the brain penetration of bedaquiline, linezolid, and pretomanid following oral administration.

**Conclusion:**

Dynamic ^18^F-pretomanid PET provided holistic data on pretomanid exposures showing excellent penetration into infected lung and brain tissues. The BPaL regimen was inferior to standard TB treatment for TB meningitis. Thus, new pretomanid-containing regimens need to be developed for the treatment of MDR-TB meningitis.

**Disclosures:**

**Charles A. Peloquin, Pharm.D.**, Nothing to disclose **Alvaro A. Ordonez, MD**, **Cubresa** (Consultant)**Fujirebio Diagnostics** (Research Grant or Support) **Sanjay K. Jain, MD**, **Fujirebio Diagnostics, Inc., USA** (Research Grant or Support)**Novobiotic LLC, USA** (Research Grant or Support)**T3 Pharma, Switzerland** (Research Grant or Support) **Sanjay K. Jain, MD**, Fujirebio Diagnostics, Inc., USA (Individual(s) Involved: Self): Research Grant or Support; Novobiotic LLC, USA (Individual(s) Involved: Self): Research Grant or Support; T3 Pharma, Switzerland (Individual(s) Involved: Self): Research Grant or Support

